# A tutorial on pilot studies: the what, why and how

**DOI:** 10.1186/1471-2288-10-1

**Published:** 2010-01-06

**Authors:** Lehana Thabane, Jinhui Ma, Rong Chu, Ji Cheng, Afisi Ismaila, Lorena P Rios, Reid Robson, Marroon Thabane, Lora Giangregorio, Charles H Goldsmith

**Affiliations:** 1 Department of Clinical Epidemiology and Biostatistics, McMaster University, Hamilton ON, Canada; 2 Biostatistics Unit, St Joseph's Healthcare Hamilton, Hamilton ON, Canada; 3 Department of Medical Affairs, GlaxoSmithKline Inc., Mississauga ON, Canada; 4 Department of Medicine, Division of Gastroenterology, McMaster University, Hamilton ON, Canada; 5 Department of Kinesiology, University of Waterloo, Waterloo ON, Canada

## Abstract

Pilot studies for phase III trials - which are comparative randomized trials designed to provide preliminary evidence on the clinical efficacy of a drug or intervention - are routinely performed in many clinical areas. Also commonly know as "feasibility" or "vanguard" studies, they are designed to assess the safety of treatment or interventions; to assess recruitment potential; to assess the feasibility of international collaboration or coordination for multicentre trials; to increase clinical experience with the study medication or intervention for the phase III trials. They are the best way to assess feasibility of a large, expensive full-scale study, and in fact are an almost essential pre-requisite. Conducting a pilot prior to the main study can enhance the likelihood of success of the main study and potentially help to avoid doomed main studies. The objective of this paper is to provide a detailed examination of the key aspects of pilot studies for phase III trials including: 1) the general reasons for conducting a pilot study; 2) the relationships between pilot studies, proof-of-concept studies, and adaptive designs; 3) the challenges of and misconceptions about pilot studies; 4) the criteria for evaluating the success of a pilot study; 5) frequently asked questions about pilot studies; 7) some ethical aspects related to pilot studies; and 8) some suggestions on how to report the results of pilot investigations using the CONSORT format.

## 1. Introduction

The Concise Oxford Thesaurus [1] defines a *pilot project or study *as an *experimental, exploratory, test, preliminary, trial or try out *investigation. Epidemiology and statistics dictionaries provide similar definitions of a pilot study as a small scale

• " ...***test of the methods and procedures to be used on a larger scale ***if the pilot study demonstrates that the methods and procedures can work" [2];

• "...investigation designed to ***test the feasibility of methods and procedures ***for later use on a large scale or ***to search for possible effects and associations ***that may be worth following up in a subsequent larger study" [3].

Table 1 provides a summary of definitions found on the Internet. A closer look at these definitions reveals that they are similar to the ones above in that a pilot study is synonymous with a feasibility study intended to guide the planning of a large-scale investigation. Pilot studies are sometimes referred to as "vanguard trials" (i.e. pre-studies) intended to assess the safety of treatment or interventions; to assess recruitment potential; to assess the feasibility of international collaboration or coordination for multicentre trials; to evaluate surrogate marker data in diverse patient cohorts; to increase clinical experience with the study medication or intervention, and identify the optimal dose of treatments for the phase III trials [4]. As suggested by an African proverb from the Ashanti people in Ghana "*You never test the depth of a river with both feet*", the main goal of pilot studies is to assess feasibility so as to avoid potentially disastrous consequences of embarking on a large study - which could potentially "drown" the whole research effort.

**Table 1:** Some Adapted Definitions of Pilot Studies on the Web (Date of last access: December 22, 2009)

Definition*	Source
A trial study carried out before a research design is finalised **to assist in defining the research question **or **to test the feasibility, reliability and validity of the proposed study design**	http://www.cirem.org.uk/definitions.html

A smaller version of a study is carried out before the actual investigation is done. Researchers use information gathered in pilot studies **to refine or modify the research methodology for a study and to develop large-scale studies**	http://www.mh.state.oh.us/what-we-do/promote/research-and-evaluation/learning-lab/research-glossary.shtml

A small scale study conducted to test the plan and method of a research study.	http://www.umm.edu/nursing/docs/glossary_research_terms.pdf

A small study **carried out before a large-scale study to try out a procedure or to test a principle**	http://www.psych-sci.manchester.ac.uk/actnow/glossary/

An experimental use of a treatment in a small group of patients **to learn if it will be effective and safe on a broad scale**	http://www.lungcanceralliance.org/news/glossary.html

The **initial study examining a new method **or treatment	http://www.cdc.gov/des/consumers/resources/glossary.html#P

A small study often done **to assist the preparation of a larger, more comprehensive investigation**.	http://www.informedesign.umn.edu/Glossary.aspx?id=1952#

Small, preliminary test or trial run of an intervention, or of an evaluation activity such as an instrument or sampling procedure. The results of the pilot are **used to improve the program or evaluation procedure being piloted before it is used on a larger scale**.	http://www.nsf.gov/pubs/2005/nsf0531/nsf0531_6.pdf

*Emphasis is ours

Feasibility studies are routinely performed in many clinical areas. It is fair to say that every major clinical trial had to start with some piloting or a small scale investigation to assess the feasibility of conducting a larger scale study: critical care [5], diabetes management intervention trials [6], cardiovascular trials [7], primary healthcare [8], to mention a few.

Despite their noted importance, the reality is that pilot studies receive little or no attention in scientific research training. Few epidemiology or research textbooks cover the topic with the necessary detail. In fact, we are not aware of any textbook that dedicates a chapter on this issue - many just mention it in passing or provide a cursory coverage of the topic. The objective of this paper is to provide a detailed examination of the key aspects of pilot studies. In the next section, we narrow the focus of our definition of a pilot to phase III trials. Section 3 covers the general reasons for conducting a pilot study. Section 4 deals with the relationships between pilot studies, proof-of-concept studies, and adaptive designs, while section 5 addresses the challenges of pilot studies. Evaluation of a pilot study (i.e. how to determine if a pilot study was successful) is covered in Section 6. We deal with several frequently asked questions about pilot studies in Section 7 using a "question-and-answer" approach. Section 8 covers some ethical aspects related to pilot studies; and in Section 9, we follow the CONSORT format [9] to offer some suggestions on how to report the results of pilot investigations.

## 2. Narrowing the focus: Pilot studies for randomized studies

Pilot studies can be conducted in both quantitative and qualitative studies. Adopting a similar approach to Lancaster *et al*. [10], we focus on quantitative pilot studies - particularly those done prior to full-scale phase III trials. Phase I trials are non-randomized studies designed to investigate the pharmacokinetics of a drug (i.e. how a drug is distributed and metabolized in the body) including finding a dose that can be tolerated with minimal toxicity. Phase II trials provide preliminary evidence on the clinical efficacy of a drug or intervention. They may or may not be randomized. Phase III trials are randomized studies comparing two or more drugs or intervention strategies to assess efficacy and safety. Phase IV trials, usually done after registration or marketing of a drug, are non-randomized surveillance studies to document experiences (e.g. side-effects, interactions with other drugs, etc) with using the drug in practice.

For the purposes of this paper, our approach to utilizing pilot studies relies on the model for complex interventions advocated by the British Medical Research Council - which explicitly recommends the use of feasibility studies prior to Phase III clinical trials, but stresses the iterative nature of the processes of development, feasibility and piloting, evaluation and implementation [11].

## 3. Reasons for Conducting Pilot Studies

Van Teijlingen *et al*. [12] and van Teijlingen and Hundley [13] provide a summary of the reasons for performing a pilot study. In general, the rationale for a pilot study can be grouped under several broad classifications - process, resources, management and scientific (see also http://www.childrens-mercy.org/stats/plan/pilot.asp for a different classification):

• *Process: *This assesses the feasibility of the steps that need to take place as part of the main study. Examples include determining recruitment rates, retention rates, etc.

• *Resources: *This deals with assessing time and budget problems that can occur during the main study. The idea is to collect some pilot data on such things as the length of time to mail or fill out all the survey forms.

• *Management: *This covers potential human and data optimization problems such as personnel and data management issues at participating centres.

• *Scientific: *This deals with the assessment of treatment safety, determination of dose levels and response, and estimation of treatment effect and its variance.

Table 2 summarizes this classification with specific examples.

**Table 2:** Reasons for conducting pilot studies

Main Reason	Examples
**Process: **This assesses the feasibility of the processes that are key to the success of the main study	• Recruitment rates
	• Retention rates
	• Refusal rates
	• Failure/success rates
	• (Non)compliance or adherence rates
	• eligibility criteria
	- Is it obvious who meets and who does not meet the eligibility requirements?
	- Are the eligibility criteria sufficient or too restrictive?
	• Understanding of study questionnaires or data collection tools:
	- Do subjects provide no answer, multiple answers, qualified answers, or unanticipated answers to study questions?

**Resources: **This deals with assessing time and resource problems that can occur during the main study	• Length of time to fill out all the study forms
	• Determining capacity:
	- Will the study participants overload your phone lines or overflow your waiting room?
	• Determining process time
	- How much time does it take to mail out a thousand surveys?
	• Is the equipment readily available when and where it is needed?
	• What happens when it breaks down or gets stolen?
	• Can the software used for capturing data read and understand the data?
	• Determining centre willingness and capacity
	- Do the centres do what they committed to doing?
	- Do investigators have the time to Perform the tasks they committed to doing?
	- Are there any capacity issues at each participating centre?

**Management: **This covers potential human and data management problems	• What are the challenges that participating centres have with managing the study?
	• What challenges do study personnel have?
	• Is there enough room on the data collection form for all of the data you receive?
	• Are there any problems entering data into the computer?
	• Can data coming from different sources be matched?
	• Were any important data values forgotten about?
	• Do data show too much or too little variability?

**Scientific: **This deals with the assessment of treatment safety, dose, response, effect and variance of the effect	• Is it safe to use the study drug/intervention?
	• What is the safe dose level?
	• Do patients respond to the drug?
	• What is the estimate of the treatment effect?
	• What is the estimate of the variance of the treatment effect?

## 4. Relationships between Pilot Studies, Proof-of-Concept Studies, and Adaptive Designs

A proof-of-concept (PoC) study is defined as a clinical trial carried out to determine if a treatment (drug) is biologically active or inactive [14]. PoC studies usually use surrogate markers as endpoints. In general, they are phase I/II studies - which, as noted above, investigate the safety profile, dose level and response to new drugs [15]. Thus, although designed to inform the planning of phase III trials for registration or licensing of new drugs, PoC studies may not necessarily fit our restricted definition of pilot studies aimed at assessing feasibility of phase III trials as outlined in Section 2.

An adaptive trial design refers to a design that allows modifications to be made to a trial's design or statistical procedures *during *its conduct, with the purpose of efficiently identifying clinical benefits/risks of new drugs or to increase the probability of success of clinical development [16]. The adaptations can be prospective (e.g. stopping a trial early due to safety or futility or efficacy at interim analysis); concurrent (e.g. changes in eligibility criteria, hypotheses or study endpoints) or retrospective (e.g. changes to statistical analysis plan prior to locking database or revealing treatment codes to trial investigators or patients). Piloting is normally built into adaptive trial designs by determining a *priori *decision rules to guide the adaptations based on cumulative data. For example, data from interim analyses could be used to refine sample size calculations [17,18]. This approach is routinely used in internal pilot studies - which are primarily designed to inform sample size calculation for the main study, with recalculation of the sample size as the key adaptation. Unlike other phase III pilots, an internal pilot investigation does not usually address any other feasibility aspects - because it is essentially part of the main study [10,19,20]..

Nonetheless, we need to emphasize that whether or not a study is a pilot, depends on its objectives. An adaptive method is used as a strategy to reach that objective. Both a pilot and a non-pilot could be adaptive.

## 5. Challenges of and Common Misconceptions about Pilot Studies

Pilot studies can be very informative, not only to the researchers conducting them but also to others doing similar work. However, many of them never get published, often because of the way the results are presented [13]. Quite often the emphasis is wrongly placed on statistical significance, not on feasibility - which is the main focus of the pilot study. Our experience in reviewing submissions to a research ethics board also shows that most of the pilot projects are not well designed: i.e. there are no clear feasibility objectives; no clear analytic plans; and certainly no clear criteria for success of feasibility.

In many cases, pilot studies are conducted to generate data for sample size calculations. This seems especially sensible in situations where there are no data from previous studies to inform this process. However, it can be dangerous to use pilot studies to estimate treatment effects, as such estimates may be unrealistic/biased because of the limited sample sizes. Therefore if not used cautiously, results of pilot studies can potentially mislead sample size or power calculations [21] -- particularly if the pilot study was done to see if there is likely to be a treatment effect in the main study. In section 6, we provide guidance on how to proceed with caution in this regard.

There are also several misconceptions about pilot studies. Below are some of the common reasons that researchers have put forth for calling their study a pilot.

The first common reason is that a pilot study is a small single-centre study. For example, researchers often state lack of resources for a large multi-centre study as a reason for doing a pilot. The second common reason is that a pilot investigation is a small study that is similar in size to someone else's published study. In reviewing submissions to a research ethics board, we have come across sentiments such as

• *So-and-so did a similar study with 6 patients and got statistical significance - ours uses 12 patients (double the size)!*

• *We did a similar pilot before (and it was published!)*

The third most common reason is that a pilot is a small study done by a student or an intern - which can be completed quickly and does not require funding. Specific arguments include

• *I have funding for 10 patients only;*

• *I have limited seed (start-up) funding;*

• *This is just a student project!*

• *My supervisor (boss) told me to do it as a pilot*.

None of the above arguments qualifies as sound reasons for calling a study a pilot. A study should only be conducted if the results will be informative; studies conducted for the reasons above may result in findings of limited utility, which would be a waste of the researchers' and participants' efforts. The focus of a pilot study should be on assessment of feasibility, unless it was powered appropriately to assess statistical significance. Further, there is a vast number of poorly designed and reported studies. Assessment of the quality of a published report may be helpful to guide decisions of whether the report should be used to guide planning or designing of new studies. Finally, if a trainee or researcher is assigned a project as a pilot it is important to discuss how the results will inform the planning of the main study. In addition, clearly defined feasibility objectives and rationale to justify piloting should be provided.

### Sample Size for Pilot Studies

In general, sample size calculations may not be required for some pilot studies. It is important that the sample for a pilot be representative of the target study population. It should also be based on the same inclusion/exclusion criteria as the main study. As a rule of thumb, a pilot study should be large enough to provide useful information about the aspects that are being assessed for feasibility. Note that *PoC studies *require sample size estimation based on surrogate markers [22], but they are usually not powered to detect meaningful differences in clinically important endpoints. The sample used in the pilot may be included in the main study, but caution is needed to ensure the key features of the main study are preserved in the pilot (e.g. blinding in randomized controlled trials). We recommend if any pooling of pilot and main study data is considered, this should be planned beforehand, described clearly in the protocol with clear discussion of the statistical consequences and methods. The goal is to avoid or minimize the potential bias that may occur due to multiple testing issues or any other opportunistic actions by investigators. In general, pooling when done appropriately can increase the efficiency of the main study [23].

As noted earlier, a carefully designed pilot study may be used to generate information for sample size calculations. Two approaches may be helpful to optimize information from a pilot study in this context: First, consider eliciting qualitative data to supplement the quantitative information obtained in the pilot. For example, consider having some discussions with clinicians using the approach suggested by Lenth [24] to illicit additional information on possible effect size and variance estimates. Second, consider creating a sample size table for various values of the effect or variance estimates to acknowledge the uncertainty surrounding the pilot estimates.

In some cases, one could use a **confidence interval [CI] approach **to estimate the sample size required to establish feasibility. For example, suppose we had a pilot trial designed primarily to determine adherence rates to the standardized risk assessment form to enhance venous thromboprophylaxis in hospitalized patients. Suppose it was also decided *a priori *that the criterion for success would be: the main trial would be '*feasibl*e' if the risk assessment form is completed for ≥ 70% of eligible hospitalized patients.

Using a 95% CI for the proportion of eligible patients who complete the assessment form, a margin of error (ME) of 0.05, a lower bound of this CI of 0.70, and an expected completion rate of 75% based on an educated guess, the required sample for the pilot study would be at least 75 patients. This calculation is based on a common formula for obtaining a 95% CI for a single proportion: *p *± 1.96  where "*p*" is the prior estimate of the proportion of interest and "*n*" is the sample size.

## 6. How to Interpret the Results of a Pilot Study: Criteria for Success

It is always important to state the criteria for success of a pilot study. The criteria should be based on the primary feasibility objectives. These provide the basis for interpreting the results of the pilot study and determining whether it is feasible to proceed to the main study. In general, the outcome of a pilot study can be one of the following: (i) *Stop *- main study not feasible; (ii) *Continue, but modify protocol *- feasible with modifications; (iii) *Continue without modifications, but monitor closely *- feasible with close monitoring and (iv) *Continue without modifications *- feasible as is.

For example, the Prophylaxis of Thromboembolism in Critical Care Trial (PROTECT) was designed to assess the feasibility of a large-scale trial with the following criteria for determining success [25]:

• *98.5% of patients had to receive study drug within 12 hours of randomization;*

• *91.7% of patients had to receive every scheduled dose of the study drug in a blinded manner;*

• *90% or more of patients had to have lower limb compression ultrasounds performed at the specified times; and *

• *> 90% of necessary dose adjustments had to have been made appropriately in response to pre-defined laboratory criteria*.

In a second example, the PeriOperative Epidural Trial (POET) Pilot Study was designed to assess the feasibility of a large, multicentre trial with the following criteria for determining success [26]:

• *one subject per centre per week (i.e., 200 subjects from four centres over 50 weeks) can be recruited*;

• *at least 70% of all eligible patients can be recruited*;

• *no more than 5% of all recruited subjects crossed over from one modality to the other; and*

• *complete follow-up in at least 95% of all recruited subjects*.

## 7. Frequently asked questions about pilot studies

In this Section, we offer our thoughts on some of the frequently asked questions about pilot studies. These could be helpful to not only clinicians and trainees, but to anyone who is interested in health research.

• ***Can I publish the results of a pilot study?***

- Yes, every attempt should be made to publish.

• ***Why is it important to publish the results of pilot studies?***

- To provide information about feasibility to the research community to save resources being unnecessarily spent on studies that may not be feasible. Further, having such information can help researchers to avoid duplication of efforts in assessing feasibility.

- Finally, researchers have an ethical and scientific obligation to attempt publishing the results of every research endeavor. However, our focus should be on feasibility goals. Emphasis should not be placed on statistical significance when pilot studies are not powered to detect minimal clinically important differences. Such studies typically do not show statistically significant results - remember that underpowered studies (with no statistically significant results) are inconclusive, not negative since "no evidence of effect" is not "evidence of no effect" [27].

• ***Can I combine data from a pilot with data from the main study?***

- Yes, provided the sampling frame and methodologies are the same. This can increase the efficiency of the main study - see Section 5.

• ***Can I combine the results of a pilot with the results of another study or in a meta-analysis?***

- Yes, provided the sampling frame and methodologies are the same.

- No, if the main study is reported and it includes the pilot study.

• ***Can the results of the pilot study be valid on their own, without existence of the main study***

- Yes, if the results show that it is not feasible to proceed to the main study or there is insufficient funding.

• ***Can I apply for funding for a pilot study?***

- Yes. Like any grant, it is important to justify the need for piloting.

- The pilot has to be placed in the context of the main study.

• ***Can I randomize patients in a pilot study?***

- Yes. For a phase III pilot study, one of the goals could be to assess how a randomization procedure might work in the main study or whether the idea of randomization might be acceptable to patients [10]. In general, it is always best for a pilot to maintain the same design as the main study.

• ***How can I use the information from a pilot to estimate the sample size?***

- Use with caution, as results from pilot studies can potentially mislead sample size calculations.

- Consider supplementing the information with qualitative discussions with clinicians - see section 5; and

- Create a sample size table to acknowledge the uncertainty of the pilot information - see section 5.

• ***Can I use the results of a pilot study to treat my patients?***

- Not a good idea!

- Pilot studies are primarily for assessing feasibility.

• ***What can I do with a failed or bad pilot study?***

- No study is a complete failure; it can always be used as bad example! However, it is worth making clear that a pilot study that shows the main study is not likely to be feasible is not a failed (pilot) study. In fact, it is a success - because you avoided wasting scarce resources on a study destined for failure!

## 8. Ethical Aspects of Pilot Studies

Halpern *et al*. [28] stated that conducting underpowered trials is unethical. However, they proposed that underpowered trials are ethical in two situations: (i) small trials of interventions for rare diseases -- which require documenting explicit plans for including results with those of similar trials in a prospective meta-analysis; (ii) early-phase trials in the development of drugs or devices - provided they are adequately powered for defined purposes other than randomized treatment comparisons. Pilot studies of phase III trials (dealing with common diseases) are not addressed in their proposal. It is therefore prudent to ask: *Is it ethical to conduct a study whose feasibility can not be guaranteed (i.e. with a high probability of success)?*

It seems unethical to consider running a phase III study without having sufficient data or information about the feasibility. In fact, most granting agencies often require data on feasibility as part of their assessment of the scientific validity for funding decisions.

There is however one important ethical aspect about pilot studies that has received little or no attention from researchers, research ethics boards and ethicists alike. This pertains to the issue of the obligation that researchers have to patients or participants in a trial to disclose the feasibility nature of pilot studies. This is essential given that some pilot studies may not lead to further studies. A review of the commonly cited research ethics guidelines - the Nuremburg Code [29], Helsinki Declaration [30], the Belmont Report [31], ICH Good Clinical Practice [32], and the International Ethical Guidelines for Biomedical Research Involving Human Subjects [33] - shows that pilot studies are not addressed in any of these guidelines. Canadian researchers are also encouraged to follow the Tri-Council Policy Statement (TCPS) [34] - it too does not address how pilot studies need to be approached. It seems to us that given the special nature of feasibility or pilot studies, the disclosure of their purpose to study participants requires special wording - that informs them of the definition of a pilot study, the feasibility objectives of the study, and also clearly defines the criteria for success of feasibility. To fully inform participants, we suggest using the following wording in the consent form:

"*The overall purpose of this pilot study is to assess the feasibility of conducting a large study to [state primary objective of the main study]. A feasibility or pilot study is a study that... [state a general definition of a feasibility study]. The specific feasibility objectives of this study are ... [state the specific feasibility objectives of the pilot study]. We will determine that it is feasible to carry on the main study if ... [state the criteria for success of feasibility]*."

## 9. Recommendation for Reporting the Results of Pilot Studies

Adopted from the CONSORT Statement [9], Table 3 provides a checklist of items to consider including in a report of a pilot study.

**Table 3:** Pilot Study - Checklist: Items to include when reporting a pilot study

PAPER SECTION	Item	Descriptor	Reported on Page #
**TITLE *and *ABSTRACT**	1	Does the title or abstract indicate that the study is a "pilot"?	

**INTRODUCTION**			
Background	2	Scientific background for the main study and explanation of rationale for assessing feasibility through piloting	

**METHODS**			
Participants and setting	3	• Eligibility criteria for participants in the pilot study (these should be the same as in the main study -- if different, state the differences)	
		• The settings and locations where the data were collected	

Interventions	4	Provide precise details of the interventions intended for each group and how and when they were actually administered (if applicable) -- state clearly if any aspects of the intervention are assessed for feasibility	

Objectives	5	• Specific scientific objectives and hypotheses for the main study	
		• Specific feasibility objectives	

Outcomes	6	• Clearly defined primary and secondary outcome measures for the main study	
		• Clearly define the feasibility outcomes and how they were operationalized -- these should include key elements such as recruitment rates, consent rates, completion rates, variance estimates, etc	

Sample size	7	Describe how sample size was determined	
		• In general for a pilot of a phase III trial, there is no need for a formal sample size calculation. However, confidence interval approach may be used to calculate and justify the sample size based on key feasibility objective(s).	

Feasibility Criteria	8	Clearly describe the criteria for assessing success of feasibility -- these should be based on the feasibility objectives	

Statistical Methods	9	Describe the statistical methods for the analysis of primary and secondary feasibility outcomes	

Ethical Aspects	10	• State whether the study received research ethics approval	
		• State how informed consent was handled -- given the feasibility nature of the study	

**RESULTS**			
Participant flow	11	Flow of participants through each stage (a flow-chart is strongly recommended).	
		• Describe protocol deviations from pilot study as planned, together with reasons	
		• State the number of exclusions at each stage and reasons for exclusions	

Recruitment	12	Report the dates defining the periods of recruitment and follow-up	

Baseline data	13	Report the baseline demographic and clinical characteristics of the participants	

Outcomes and estimation	14	For each primary and secondary feasibility outcome, report the point estimate of effect and its precision (*e.g*., 95% confidence interval [CI]) -- if applicable	

**DISCUSSION**			
Interpretation	15	Interpretation of the results should focus on feasibility, taking into account	
		• the stated criteria for success of feasibility;	
		• study hypotheses, sources of potential bias or imprecision -- given the feasibility nature of the study	
		• the dangers associated with multiplicity of analyses and outcomes	

Generalizability	16	Generalizability (external validity) of the feasibility. State clearly what modifications in the design of the main study (if any) would be necessary to make it feasible	

Overall evidence of feasibility	17	General interpretation of the results in the context of current evidence of feasibility	
		• Focus should be on feasibility	

### Title and abstract

#### Item #1: The title or abstract should indicate that the study is a "pilot" or "feasibility"

As a number one summary of the contents of any report, it is important for the title to clearly indicate that the report is for a pilot or feasibility study. This would also be helpful to other researchers during electronic information search about feasibility issues. Our quick search of PUBMED [on July 13, 2009], using the terms "pilot" OR "feasibility" OR "proof-of-concept" for revealed 24423 (16%) hits of studies that had these terms in the title or abstract compared with 149365 hits that had these terms anywhere in the text.

### Background

#### Item #2: Scientific background for the main study and explanation of rationale for assessing feasibility through piloting

The rationale for initiating a pilot should be based on the need to assess feasibility for the main study. Thus, the background of the main study should clearly describe what is known or not known about important feasibility aspects to provide context for piloting.

### Methods

#### Item #3: Participants and setting of the study

The description of the inclusion-exclusion or eligibility criteria for participants should be the same as in the main study. The settings and locations where the data were collected should also be clearly described.

#### Item #4: Interventions

Precise details of the interventions intended for each group and how and when they were actually administered (if applicable) - state clearly if any aspects of the intervention are assessed for feasibility.

#### Item #5: Objectives

State the specific scientific primary and secondary objectives and hypotheses for the main study and the specific feasibility objectives. It is important to clearly indicate the feasibility objectives as the primary focus for the pilot.

#### Item #6: Outcomes

Clearly define primary and secondary outcome measures for the main study. Then, clearly define the feasibility outcomes and how they were operationalized - these should include key elements such as recruitment rates, consent rates, completion rates, variance estimates, etc. In some cases, a pilot study may be conducted with the aim to determine a suitable (clinical or surrogate) endpoint for the main study. In such a case, one may not be able to define the primary outcome of the main study until the pilot is finished. However, it is important that determining the primary outcome of the main study be clearly stated as part of feasibility outcomes.

#### Item #7: Sample Size

Describe how sample size was determined. If the pilot is a proof-of-concept study, is the sample size calculated based on primary/key surrogate marker(s)? In general if the pilot is for a phase III study, there may be no need for a formal sample size calculation. However, the confidence interval approach may be used to calculate and justify the sample size based on key feasibility objective(s).

#### Item #8: Feasibility criteria

Clearly describe the criteria for assessing success of feasibility - these should be based on the feasibility objectives.

#### Item #9: Statistical Analysis

Describe the statistical methods for the analysis of primary and secondary feasibility outcomes.

#### Item #10: Ethical Aspects

State whether the study received research ethics approval. Describe how informed consent was handled - given the feasibility nature of the study.

### Results

#### Item #11: Participant Flow

Describe the flow of participants through each stage of the study (use of a flow-diagram is strongly recommended -- see CONSORT [9] for a template). Describe protocol deviations from pilot study as planned with reasons for deviations. State the number of exclusions at each stage and corresponding reasons for exclusions.

#### Item #12: Recruitment

Report the dates defining the periods of recruitment and follow-up.

#### Item #13: Baseline Data

Report the baseline demographic and clinical characteristics of the participants.

#### Item #14: Outcomes and Estimation

For each primary and secondary feasibility outcomes, report the point estimate of effect and its precision (*e.g*., 95% CI) - if applicable.

### Discussion

#### Item # 15: Interpretation

Interpretation of the results should focus on feasibility, taking into account the stated criteria for success of feasibility, study hypotheses, sources of potential bias or imprecision (given the feasibility nature of the study) and the dangers associated with multiplicity - repeated testing on multiple outcomes.

#### Item #16: Generalizability

Discuss the generalizability (external validity) of the feasibility aspects observed in the study. State clearly what modifications in the design of the main study (if any) would be necessary to make it feasible.

#### Item #17: Overall evidence of feasibility

Discuss the general results in the context of overall evidence of feasibility. It is important that the focus be on feasibility.

## 9. Conclusions

Pilot or vanguard studies provide a good opportunity to assess feasibility of large full-scale studies. Pilot studies are the best way to assess feasibility of a large expensive full-scale study, and in fact are an almost essential pre-requisite. Conducting a pilot prior to the main study can enhance the likelihood of success of the main study and potentially help to avoid doomed main studies. Pilot studies should be well designed with clear feasibility objectives, clear analytic plans, and explicit criteria for determining success of feasibility. They should be used cautiously for determining treatment effects and variance estimates for power or sample size calculations. Finally, they should be scrutinized the same way as full scale studies, and every attempt should be taken to publish the results in peer-reviewed journals.

## Competing interests

The authors declare that they have no competing interests.

## Authors' contributions

LT drafted the manuscript. All authors reviewed several versions of the manuscript, read and approved the final version.

## Pre-publication history

The pre-publication history for this paper can be accessed here:

http://www.biomedcentral.com/1471-2288/10/1/prepub
