# Correction to: A tutorial on pilot studies: the what, why and how

**DOI:** 10.1186/s12874-023-01880-1

**Published:** 2023-03-11

**Authors:** Lehana Thabane, Jinhui Ma, Rong Chu, Ji Cheng, Afisi Ismaila, Lorena P Rios, Reid Robson, Marroon Thabane, Lora Giangregorio, Charles H Goldsmith

**Affiliations:** 1 Department of Clinical Epidemiology and Biostatistics, McMaster University, Hamilton, ON Canada; 2 Biostatistics Unit, St Joseph’s Healthcare Hamilton, Hamilton, ON Canada; 3 Department of Medical Affairs, GlaxoSmithKline Inc, Mississauga, ON Canada; 4 Department of Medicine, Division of Gastroenterology, McMaster University, Hamilton, ON Canada; 5 Department of Kinesiology, University of Waterloo, Waterloo, ON Canada

**Correction to: BMC Medical Research Methodology 10, 1 (2010)**

10.1186/1471-2288-10-1

Following publication of the original article [1], the authors would like to correct the number of sample size in the fourth paragraph under the heading **Sample Size for Pilot Studies**.

The sentence currently reads:

Using a 95% CI for the proportion of eligible patients who complete the assessment form, a margin of error (ME) of 0.05, a lower bound of this CI of 0.70, and an expected completion rate of 75% based on an educated guess, the required sample for the pilot study would be at least 75 patients.

The sentence should read:

Using a 95% CI for the proportion of eligible patients who complete the assessment form, a margin of error (ME) of 0.05, a lower bound of this CI of 0.70, and an expected completion rate of 75% based on an educated guess, the required sample for the pilot study would be at least 289 patients.

The original article [1] has been corrected.
